# Conspecific pollen advantage mediated by the extragynoecial compitum and its potential to resist interspecific reproductive interference between two *Sagittaria* species

**DOI:** 10.3389/fpls.2022.956193

**Published:** 2022-07-22

**Authors:** Cai-Hong Fei, Sha-Sha Tang, Shu-He Shang, Jie Dai, Xin-Yi Wang, Shuai Wang, Wei-Qi Liu, Xiao-Fan Wang

**Affiliations:** ^1^College of Life Sciences, Wuhan University, Wuhan, China; ^2^College of Life Science, Hengyang Normal University, Hengyang, China

**Keywords:** conspecific pollen advantage, extragynoecial compitum, reproductive interference, heterospecific pollen deposition, interspecific seed discounting, *Sagittaria*

## Abstract

The extragynoecial compitum formed by the incomplete fusion of carpel margins, while allowing intercarpellary growth of pollen tubes in apocarpous angiosperms, may also increase the risk of reproductive interference caused by heterospecific pollen (HP) deposition. In *Sagittaria*, congeneric HP tubes grow via different paths and enter the ovules later than conspecific pollen (CP) tubes. However, it is unclear how the growth advantage of the CP tube helps ensure reproductive success when HP is deposited on the stigmas. We performed molecular characterization of interspecies-pollinated seeds to examine the consequences of interspecific pollen deposition between *Sagittaria pygmaea* and *S. trifolia*. We also conducted CP–HP (1:1) mixed pollination and delayed CP pollination treatments to explore the seed-siring abilities of CP and HP. Our results showed that although HP could trigger the development of fruits, the interspecies-pollinated seeds contained partially developed embryos and could not germinate. More than 70% of the embryos in these seeds were molecularly identified as hybrids of both species, suggesting that HP tubes could enter the ovules and fertilize the egg cells. Moreover, CP could sire more offspring (≥70%) after the CP–HP (1:1) mixed pollination treatment, even when HP reached the stigma 0.5–1 h earlier than CP (≥50%). Following adequate CP vs. HP (1:1) pollination on carpels on two sides of the apocarpous gynoecium, both species produced > 70% conspecific seeds, indicating that the CP tubes could occupy ovules that should be occupied by HP via the extragynoecial compitum. Our results reveal that in *Sagittaria*, pollen deposition from co-existing congeneric heterospecies leads to interspecific seed discounting. However, the CP advantage mediated by the extragynoecial compitum is an effective strategy to mitigate the effects of interspecific pollen deposition. This study improves our understanding of how apocarpous angiosperms with an extragynoecial compitum can maintain species stability and mitigate the negative reproductive interference effect from sympatrically distributed related species.

## Introduction

When pollinators visit co-flowering and co-occurring plant species alternately, pollen from one species may be transferred to another (Morales and Traveset, 2008). Interspecific pollen transfer is often considered a form of reproductive interference that reduces the reproductive success of at least one species involved (Waser, 1978; Campbell, 1985; Mitchell et al., 2009; Muchhala and Thomson, 2012; Moreira-Hernández and Muchhala, 2019). Recent evidences suggest that interspecific pollen transfer is common in natural communities and that heterospecific pollen (HP) loads can vary considerably in size and composition (Ashman and Arceo-Gomez, 2013; Fang and Huang, 2013; Emer et al., 2015; Tur et al., 2016; Lopes et al., 2021). HP can reduce the reproductive success of recipient plants in different ways, such as stigma clogging (Galen and Gregory, 1989), stigma closure (Waser and Fugate, 1986), pollen allelopathy (Thomson et al., 1982), and stylar clogging (Randall and Hilu, 1990). Furthermore, HP deposition on closely related species is more likely to cause the abortion of seeds or whole fruits (Wang and Cruzan, 1998; Fishman and Wyatt, 1999; Wolf et al., 2001; Montgomery et al., 2010; Liao et al., 2019), failure of seed germination (Natalis and Wesselingh, 2012), or production of unfit or sterile offspring (Goodwillie and Ness, 2013), as stigmas of closely related species usually provide a relatively suitable environment for pollen germination than distantly related ones (Martin, 1970).

Intercarpellary pollen-tube growth is common in several apocarpous angiosperms, including *Illicium*, *Kadsura*, *Schisandra*, *Sedum*, *Sagittaria*, and *Ranalisma* (Williams et al., 1993; Wang et al., 2002, 2006, 2012; Lyew et al., 2007; Du and Wang, 2012; Huang and Wang, 2017). It has been reported that a number of apocarpous angiosperms have an extragynoecial compitum that allows pollen tubes to grow between the free carpels of the gynoecium and thereby may have the potential to increase fruit/seed set (Endress, 1980, 1982, 1995; Endress and Igersheim, 1997, 2000; Renner et al., 1997; Lyew et al., 2007; Endress and Doyle, 2009; Wang et al., 2012). In some apocarpous species, the extragynoecial compitum is formed by the incomplete fusion of the carpel margins; this leaves an opening that allows pollen tubes to exit the ovary to reach unfertilized ovules in other carpels after penetrating the stigma surface (Williams et al., 1993; Wang et al., 2002; Wang et al., 2006; Lyew et al., 2007; Du and Wang, 2012; Wang et al., 2012; Huang et al., 2014). However, the incomplete fusion of the carpel margins may also provide more opportunities for HP tubes to enter ovaries and occupy ovules when HP—especially those from closely related species—are deposited on stigmas. For example, Lyu et al. (2017) found that HP tubes of congeneric plants can enter the ovules in adjacent carpels through the basal opening of the carpel for fertilization. Consequently, the incomplete fusion of the carpel margins can increase the risk of reproductive interference. Since the discovery of extragynoecial compitum and intercarpellary pollen-tube growth in apocarpous angiosperms, research has focused on their potential to improve the quantity and quality of offspring. Wang et al. (2002) demonstrated that in *Sagittaria potamogetifolia*, the reallocation of pollen tubes among pistils in the gynoecium could lead to the fertilization of unpollinated ovules. Du and Wang (2012) found that the intercarpellary growth of pollen tubes could significantly increase the fruit set after comparing the developed carpel ratio and pollinated carpel ratio in flowers from natural populations in *Schisandra sphenanthera*. In contrast, studies on the potential risks in terms of reproductive interference are lacking.

Conspecific pollen (CP) may have some advantages over HP, which may mitigate the risk of reproductive interference caused by HP deposition under natural conditions. Previous research has shown that CP achieves more successful ovule fertilization via faster pollen germination, faster pollen tube growth, or an increased ability to reach the micropyle and complete fertilization (Howard, 1999; Chapman et al., 2005; Brock, 2009; Montgomery et al., 2010; Zhong et al., 2019). Lyu et al. (2017) compared the growth patterns of CP tubes and HP tubes after controlled pollination between *Sagittaria pygmaea* and *S. trifolia*, and found that CP tubes tended to directly enter the ovules, whereas HP tubes preferred passing through the basal opening of the carpel to enter ovules in other carpels; the resulting HP tubes reached the ovule with a delay of approximately 1 h. However, although the above experiments reveal a growth advantage for CP tubes in *S. pygmaea* and *S. trifolia*, it cannot be concluded that this advantage can reduce reproductive interference caused by HP deposition, because previous studies in other taxa have shown that CP tube growth advantage does not always lead to conspecific seed-siring advantage. Diaz and Macnair (1999) and Song et al. (2002) found agreement between CP tube growth advantage and conspecific seed-siring advantage in *Mimulus guttatus* and rice. However, Montgomery et al. (2010) found that although *Silene dioica* showed a conspecific advantage for pollen-tube elongation, it did not show a conspecific siring advantage after mixed-species pollinations. Thus, mixed pollination experiments are necessary to determine whether a conspecific seed-siring advantage exists in both *S. pygmaea* and *S. trifolia*.

Since the discovery of intercarpellary pollen-tube growth, its role in improving fruit or seed sets has received considerable research attention. However, the question of how plants with an extragynoecial compitum maintain interspecific isolation and avoid reproductive interference has been neglected. The discovery of the growth advantage of CP tubes after interspecific pollination in *Sagittaria* is exciting in this regard. As such, the following questions deserve further study: (1) does the incomplete fusion of carpel margins lead to more severe reproductive interference following pollen deposition from closely related species? and (2) is the growth advantage of CP tubes mediated by different growth paths, thus effectively avoiding the effect of interspecific pollen deposition? To answer these questions, we performed hand-pollination experiments in two *Sagittaria* species (*S. pygmaea* and *S. trifolia*) and used molecular analysis to verify the paternity of the seeds produced. The aim of this study was to examine the potential risk of interspecific pollen deposition in sympatric apocarpous species and elucidate the role of the growth advantage of CP in maintaining species stability and ensuring reproductive success.

## Materials and methods

### Plant materials and study site

*Sagittaria pygmaea* and *S. trifolia* (Alismataceae) are monoecious aquatic herbs widely distributed in Asia that are often found growing around marshes, wetlands, and rice fields. Both species are field weeds in the main rice-growing areas in China, and their flowering period is from May to November (Chen, 1989). Both male and female flowers bloom in the morning and are only open for a single day. Female flowers contain a gynoecium with numerous carpels (approximately 100 in *S. pygmaea* and approximately 400 in *S. trifolia*), and each carpel has a single ovule. Both species are self-compatible and are pollinated by insects such as flies, bees, and syrphids. The pollen of both species is morphologically indistinguishable under a light microscope. By staining the pollen grains with safranin dye (1% water solution), pollen transfer can be tracked in arrays of potted plants. Using this technique, we detected interspecific pollen deposition on the stigmas of the female flowers of both species (Supplementary Figures 1, 2).

In 2018, we planted approximately 100 and 40 seedlings of *S. pygmaea* and *S. trifolia*, respectively. These seedlings were collected from monospecific populations in Wuhan, Hubei Province, China (30°28′N, 114°20′E). We cultivated the seedlings with daily watering and fertilizer application once every 2 weeks in an outdoor experimental site at Wuhan University (30°32′31″N, 114°22′2#x2033;E).

### Examination of pollen tube growth

To examine whether HP tubes can grow into ovules and whether their growth path differs from that of CP tubes, we monitored the routes of pollen tube growth. In total, we collected 5–6 female flowers subjected to intraspecific and interspecific crosses with *S. pygmaea* and *S. trifolia* as maternal parents, respectively. After 1.5 and 3 h, each female flower was fixed with formalin–acetic acid–alcohol (FAA) (5:5:90 formalin–acetic acid–70% ethanol, v/v), softened with NaOH (10%) until most of the tissue was transparent, rinsed with distilled water, and then stained with aniline blue (0.1% in 1/30 mol l^–1^ K_3_PO_4_) for 2 h (Wang et al., 2002). The stained styles and ovaries were mounted on slides and observed under a fluorescence microscope (BX43F; Olympus, Tokyo, Japan).

### Seed set and viability measurement

We conducted hand-pollination experiments to investigate the effect of interspecific pollen deposition between *S. pygmaea* and *S. trifolia*. Pollinations were carried out from August to September in 2019 and 2020. We used approximately 140 individuals (approximately 100 *S. pygmaea* and 40 *S. trifolia*) as pollen recipients, and all the plants available during the pollination treatments were used as pollen donors. Each newly opened female flower of the day was randomly assigned one of the two pollination treatments. In total, for *S. pygmaea*, 30 female flowers were subjected to interspecific hand pollination and 32 female flowers to intraspecific hand pollination. For *S. trifolia*, 15 female flowers were subjected to interspecific hand pollination and 15 female flowers to intraspecific hand pollination.

For each pollination treatment, pollen was collected and pooled from 3 to 5 plants per species by gently tapping a fully opened male flower over a Petri dish to release the pollen. Hand pollinations were performed between 07:30 and 09:30 h using a small paintbrush loaded with fresh pollen grains to evenly brush the entire gynoecium. After 2–3 weeks, we collected the mature aggregate fruits, counted the carpels per aggregate fruit, and peeled off the pericarp and seed coat to observe the morphology of embryos under a stereoscope (SZX2-ILLT; Olympus, Tokyo, Japan). In addition, mature fruits and seeds were photographed with a digital camera (DMC-GX85; Panasonic, Osaka, Japan). The height (longitudinal dimension) and width (longest transverse dimension) of the aggregate fruits (60 from *S. pygmaea* and 73 from *S. trifolia*) and achenes (60 from *S. pygmaea* and 120 from *S. trifolia*) were measured using a digital caliper and ImageJ 2.1.0 (National Institutes of Health, Bethesda, MD, United States), respectively. To compare germination rates, we sowed 700 seeds obtained from intraspecific (150 from *S. pygmaea* and 200 from *S. trifolia*) and interspecific (150 from *S. pygmaea* and 200 from *S. trifolia*) crosses. All seeds were cold-stratified, moved to a growth chamber (25°C) to germinate, and monitored for signs of germination over 14 days.

### Molecular identification of the progeny

To verify whether the unfilled seeds produced by interspecific pollination resulted from successful fertilization by HP tubes entering the ovule, we performed molecular analysis of 53 unfilled seeds (31 from *S. pygmaea* and 22 from *S. trifolia*). To determine whether the filled seeds (with U-shaped embryos) were a result of fertilization from CP tubes, we performed molecular analysis of 48 filled seeds obtained from conspecific pollination (20 in total; 10 from *S. pygmaea* and 10 from *S. trifolia*) and mixed pollination (28 in total; 16 from *S. pygmaea* and 12 from *S. trifolia*). DNA was extracted from the leaf tissue of parent plants (10 in total; five from *S. pygmaea* and five from *S. trifolia*) using a DNA extraction kit (Kangwei Century, Jiangsu, China) according to the manufacturer’s instructions. Each seed was carefully stripped of its seed coat under a stereomicroscope and transferred into 0.2-ml Eppendorf tubes for DNA extraction. Each embryo was tested as a separate sample. Because the embryos from *Sagittaria* were very small (filled embryos, approximately 0.1–0.4 mg each; unfilled embryos, approximately 0.04 mg each), we used a slightly modified Chelex-100 method (HwangBo et al., 2009) to extract the embryonic DNA. The embryos were homogenized with a pipette tip in 20 μl of 5% chelex-100 for 1 min, incubated in 56°C water for 30 min, incubated in boiling water for 8 min, and finally centrifuged at 12,000 rpm for 10 min. The supernatant was used as the template for PCR amplification.

The nuclear ribosomal internal transcribed spacer (nrITS) sequences of all sampled specimens (111) were amplified using the ITS4 and ITS5 primers (White et al., 1990). The PCR conditions were the same as those reported by Liao et al. (2016). The PCR products were purified using electrophoresis in a 1% agarose gel, from which the products were recovered with a DNA Gel Recovery Kit (Tsingke Biological Technology, Beijing, China). Considering the possible heterogeneity within ITS sequences, we performed a T-A cloning step for ITS PCR products using the pClone007 Versatile Simple Vector Kit (Tsingke Biological Technology, Beijing, China). Approximately 10–20 positive bacterial colonies were sequenced for each individual. All DNA sequences were assembled and aligned using Mega7 (Kumar et al., 2016), and variable sites and haplotypes were obtained using the DnaSP 5.0 program (Rozas et al., 2003).

### Hand-pollination to detect interspecific pollen competition

**FIGURE 1:**
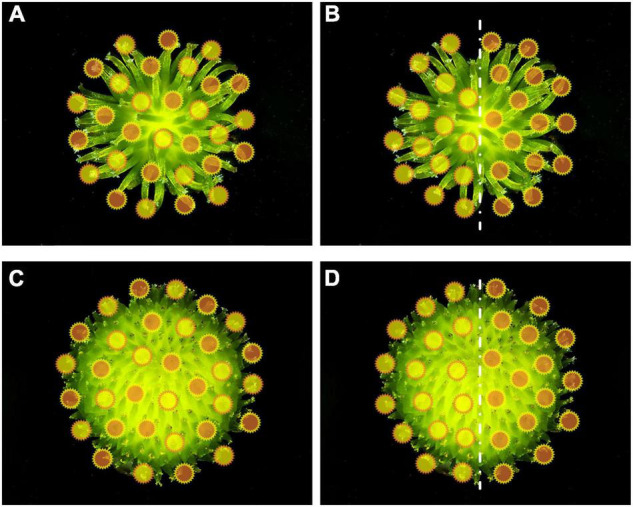
Diagram of mixed pollination **(A,C)** and half-and-half pollination **(B,D)** pollen distribution with *Sagittaria pygmaea* **(A,B)** and *S. trifolia* **(C,D)** as maternal parent, respectively. The orange circles represent *S. pygmaea* pollen and yellow circles represent *S. trifolia* pollen, but do not represent the actual pollen size.

We used hand-pollination experiments to assess whether CP had a seed-siring advantage over HP. During the flowering peak from July to September in 2019 and 2020, we performed the following four pollination treatments: (1) adequate CP and HP (1:1) mixed pollination (the total number of pollen grains was approximately 5–10 times the number of carpels); (2) inadequate CP and HP (1:1) mixed pollination (the total number of pollen grains was approximately equal to the number of carpels; (3) half-and-half pollination with CP and HP (one half of the gynoecium was pollinated with adequate CP and the other half with HP) (Figure 1); (4) delayed CP pollination (the gynoecium was pollinated first with HP, and then with CP after 0, 0.5, 1, and 2 h). Before pollination treatments, we carefully checked the stigma surface to ensure that there was no pollen deposition on the stigma.

For each pollination treatment, pollen was collected and pooled from 3 to 5 plants per species by gently tapping a fully opened male flower over a Petri dish to release the pollen. Hand pollinations were performed between 07:30 and 09:30 h using a small paintbrush loaded with fresh pollen grains. Before conducting the experiments, we verified that the brush collected ∼50–70 pollen grains per dip under a microscope in the laboratory. Therefore, the number of pollen grains applied was estimated according to the number of times the brush was used to collect pollen. We used approximately 140 individuals (approximately 100 *S. pygmaea* and 40 *S. trifolia*) as pollen recipients, and all the plants available during the pollination treatments were used as pollen donors. Each newly opened female flower of the day was randomly assigned one of the four pollination treatments. In total, for *S. pygmaea*, 30–34 flowers were selected for each treatment. For *S. trifolia*, 15–16 flowers were selected for each treatment. Approximately 2–3 weeks after pollination, we harvested the fruits, counted the carpels per aggregate fruit under a stereomicroscope, and peeled the pericarp and seed coat to count the seeds containing embryos of different shapes.

### Statistical analysis

All statistical analyses were conducted using SPSS 26.0 (Chicago, IL, United States). We used *t*-tests to compare the proportion of filled seeds sired by *S. pygmaea* and *S. trifolia* with the expected proportion based on the proportion of CP in the mixture (50%) or the proportion of CP pollinated carpels on all carpels (50%). Differences in the mean proportions of filled seeds among different treatments were analyzed with one-way ANOVA followed by a *post hoc* Scheffe’s test. Prior to these analyses, Levene’s test was used to check for homogeneity of variances, and the data were checked for normality.

## Results

### Pollen tube growth

**FIGURE 2:**
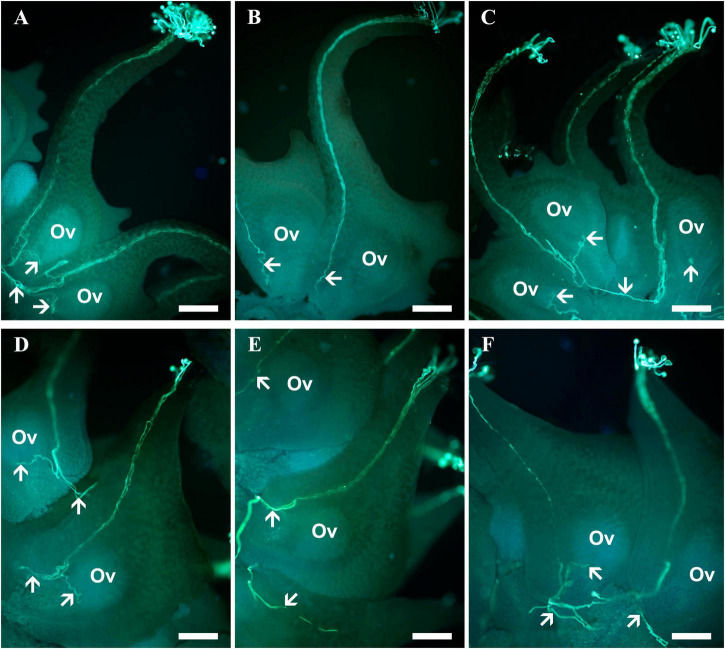
Pollen tube growth in the pistils of *Sagittaria pygmaea* and *S. trifolia* after conspecific and heterospecific pollination observed under a fluorescence microscope. **(A)** Pollen tubes of *S. pygmaea* arriving at the base of the carpel, with one of them entering into the micropyle at 1.5 h after conspecific pollination. **(B)** Pollen tubes of *S. trifolia* arriving at the base of the *S. pygmaea* carpels at 1.5 h after heterospecific pollination. **(C)** Pollen tubes of *S. trifolia* passed through the basal carpel opening enter into other unfertilized ovules at 3 h after heterospecific pollination. **(D)** Pollen tubes of *S. trifolia* arriving at the base of the carpel, with one of them entering into the micropyle at 1.5 h after conspecific pollination. **(E)** Pollen tubes of *S. pygmaea* arriving at the *S. trifolia* base of the carpel at 1.5 h after heterospecific pollination **(F)** pollen tubes of *S. pygmaea* passed through the basal carpel opening enter into other unfertilized ovules at 3 h after heterospecific pollination. White arrowheads denote pollen tubes. Ov, ovule; Scale bar: 200 μm.

*Sagittaria pygmaea* pollen germinated and successfully entered the ovule of *S. trifolia* and vice versa. However, compared to CP tubes that directly turn toward the ovules, HP tubes tended to exit from the carpel through the basal outlet and eventually enter ovules in other carpels. Pollen-tube growth routes after conspecific and heterospecific pollinations are shown in Figure 2.

### Seed set after conspecific/heterospecific pollination

**FIGURE 3:**
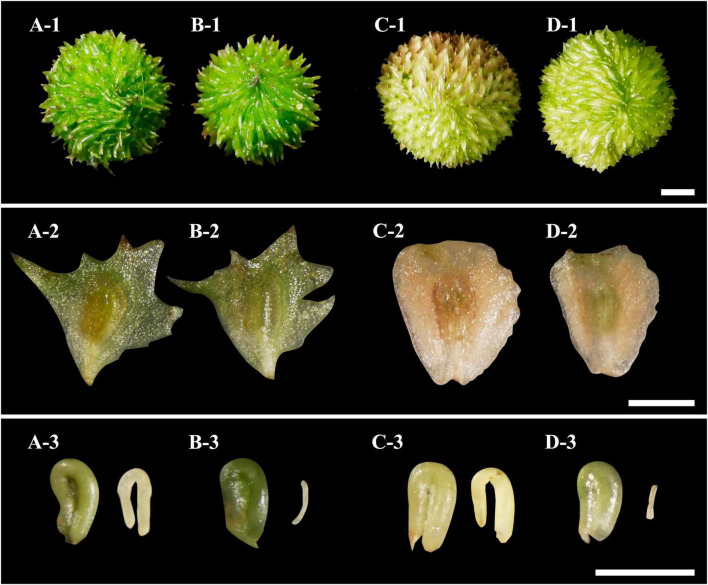
Aggregated fruits and achenes resulting from conspecific pollination and reciprocal crosses of *Sagittaria pygmaea* and *S. trifolia*, as well as images of mature seeds and embryos obtained from inside the conspecific-pollinated and interspecific-pollinated fruits. All crosses are female × male. **(A)** *S. pygmaea* × *S. pygmaea.* **(B)** *S. pygmaea* × *S. trifolia.* **(C)** *S. trifolia* × *S. trifolia.* **(D)** *S. trifolia* × *S. pygmaea.* 1, 2, and 3 represent aggregate fruits, achenes, and seeds and embryos, respectively. Scale bar: 2 mm.

All hand pollinations performed on plants of *S. pygmaea* and *S. trifolia* using CP and HP led to fruit development. Although hybrid fruits resembled intraspecific-pollinated fruits in size and morphology (Figure 3 and Supplementary Table 1), their embryos were partially developed. Stereomicroscopic observations of intraspecific crosses showed that 90.62 ± 1.96% (*S. pygmaea*) and 80.44 ± 1.15% (*S. trifolia*) of the seeds contained a well-developed U-shaped embryo (Figures 3A-3,C-3; termed filled seeds). In contrast, only 0.47 ± 0.20% (*S. pygmaea*) and 0.39 ± 0.22% (*S. trifolia*) of the seeds from interspecific crosses contained U-shaped embryos. Moreover, 78.77 ± 2.50% (*S. pygmaea*) and 61.15 ± 2.40% (*S. trifolia*) of the seeds derived from interspecific crosses contained partially developed embryos (Figures 3B-3,D-3; termed unfilled seeds). In addition, with both *S. pygmaea* and *S. trifolia* as female parents, a certain proportion of partly enlarged, or flat, carpels did not show clear signs of containing an embryo in both conspecific and heterospecific pollinations. On average, 10% (*S. pygmaea*) and 76% (*S. trifolia*) of the seeds derived from intraspecific fruits germinated, and none of the hybrid seeds germinated.

### Identification of conspecific/heterospecific offspring

The nrITS sequences had a total aligned length of 671 bp with 28 fixed nucleotide substitutions and indels, distinguishing *S. pygmaea* sequences from *S. trifolia* at the species level (Supplementary Table 2). Overall, 71.0% (*S. pygmaea*) and 77.3% (*S. trifolia*) of the seeds produced by heterospecific pollinations exhibited additive polymorphisms between the two parent species at the 28 sites (Table 1 and Supplementary Table 2). The nrITS data revealed that these seeds have nrITS sequence types of both *S. pygmaea* and *S. trifolia*, suggesting that they are the result of HP tubes entering the ovules and fertilizing the egg cells. Furthermore, 29.0% (*S. pygmaea*) and 22.7% (*S. trifolia*) of the seeds produced by heterospecific pollinations exhibited polymorphisms at some sites or no polymorphisms (Supplementary Table 2). These seeds should also be hybrid offspring, but the presence of concerted evolution of the nrITS may result in the combination of two parents in the ITS sequence of the hybrids or the complete loss of one parent’s gene (Aguilar et al., 1999). There were no polymorphisms in the nrITS sequences of all tested filled seeds (with U-shaped embryos) produced by conspecific or mixed pollination (Supplementary Table 2), suggesting that the filled seeds resulted from CP-fertilized ovules. Thus, in this study, the proportion of filled seeds (filled seeds/total seeds) was used to estimate CP reproductive success after mixed pollination.

**TABLE 1:** Molecular characterization of seeds between *Sagittaria pygmaea* and *S. trifolia* obtained from hand-pollination treatments.

Treatment	Seed type	No. of seeds for identification	No. of seeds contain ITS sequences of both *S. pygmaea* and *S. trifolia*	Percentage of hybrid seeds (%)
***S. pygmaea* (♀)**				
Conspecific pollination	Filled seeds	10	0	0.0
Mixed pollination	Filled seeds	16	0	0.0
Heterospecific pollination	Unfilled seeds	31	22	71.0
***S. trifolia* (♀)**				
Conspecific pollination	Filled seeds	10	0	0.0
Mixed pollination	Filled seeds	12	0	0.0
Heterospecific pollination	Unfilled seeds	22	17	77.3

### Seed-siring advantage of conspecific pollen over heterospecific pollen

**FIGURE 4:**
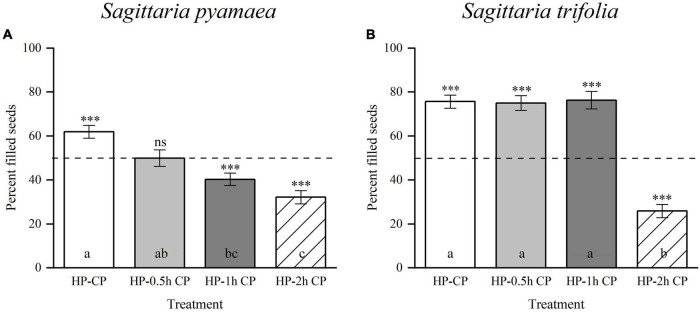
Percentages of filled seeds produced by hand-pollination experiments in which the maternal parent first received HP and later CP in *Sagittaria pygmaea* and *S. trifolia*. **(A)** *S. pygmaea* and **(B)** *S. trifolia*: HP-CP (sequence first HP then CP immediately), HP-0.5 h CP (sequence first HP 0.5 h later CP), HP-1 h CP (sequence first HP 1 h later CP) and HP-2 h CP (sequence first HP 2 h later CP). Asterisks show that the percentage of filled seeds is significantly differences from 50% (dashed line). (ns, not significant; ^***^p < 0.001). Different letters indicate significant differences among treatments (*p* < 0.05). CP, conspecific pollen, HP, heterospecific pollen.

*Sagittaria pygmaea* and *S. trifolia* produced 78.07 ± 2.01% and 83.29 ± 2.56% filled seeds in the adequate CP–HP (1:1) mixed pollination treatment, respectively, which significantly differed from the expected 50% (Table 2; *S. pygmaea*, *t* = 13.94, *p* < 0.001; *S. trifolia*, *t* = 13.00, *p* < 0.001). When the total number of CP and HP grains was reduced to be equal to the number of carpels, the proportion of filled seeds decreased but remained above 50% (Table 2; *S. pygmaea*, *t* = 6.36, *p* < 0.001; *S. trifolia*, *t* = 17.50, *p* < 0.001). These results suggest an advantage of CP for seed-siring. In the half-and-half pollination treatment with CP and HP, the proportion of filled seeds was 70.95 ± 1.69% and 74.94 ± 1.21% in *S. pygmaea* and *S. trifolia*, respectively, which were significantly higher than the expected value of 50% based on the number of pollinated carpels (Table 2; *S. pygmaea*, *t* = 12.41, *p* < 0.001; *S. trifolia*, *t* = 20.60, *p* < 0.001). These results suggest that the CP advantage is associated with intercarpellary pollen tube growth. When HP was applied before CP, the proportion of filled seeds decreased with increasing time interval between HP application and CP application at the stigma and the proportion of filled seeds differed significantly among the four treatments [Figure 4; one-way ANOVA, *S. pygmaea*, *F*_(3,120)_ = 16.570, *p* < 0.001; *S. trifolia, F*_(3,57)_ = 54.517, *p* < 0.001]. In *S. pygmaea*, the proportion of filled seeds was close to 50% (49.93 ± 3.74%) when CP application was delayed by 0.5 h. However, in *S. trifolia*, the proportion of filled seeds was maintained at 76.27 ± 4.01% even when CP application was delayed by 1 h. In addition, compared with the CP–HP (1:1) mixed pollination treatment with adequate amounts of pollen grains, the HP–CP successive pollination treatment showed a low proportion of filled seeds (*S. pygmaea*, 61.91 ± 2.96%, *t* = 4.51, *p* < 0.001; *S. trifolia*, 75.64 ± 2.92%, *t* = 1.74, *p* = 0.094). Therefore, the seed-siring advantage of CP may be underestimated in these treatments due to the spatial barrier created by HP on the stigma.

**TABLE 2:** Percentages of each mature seed phenotype resulting from different hand-pollination treatments in *Sagittaria pygmaea* and *S. trifolia.*

Treatment	N	Average carpel number per aggregate fruit	Carpels without seeds/Total carpels (%)	Carpels with unfilled seeds/Total carpels (%)	Carpels with filled seeds/Total carpels (%)	Filled seeds/Total seeds (%)
***S. pygmaea* (♀)**						
CP only	32	128.22 ± 6.21	6.07 ± 1.83	3.31 ± 0.70	90.62 ± 1.96	96.40 ± 0.76
HP only	30	101.23 ± 3.96	20.76 ± 2.45	78.77 ± 2.50	0.47 ± 0.20	0.68 ± 0.30
Mix (adequate)	30	105.40 ± 6.13	14.20 ± 2.83	18.34 ± 1.61	67.46 ± 2.94	78.07 ± 2.01***
Mix (inadequate)	34	93.38 ± 5.11	30.87 ± 3.39	20.52 ± 2.05	48.61 ± 3.51	68.87 ± 2.97***
Half-and-half	34	125.94 ± 4.30	11.65 ± 1.88	25.55 ± 1.51	62.80 ± 2.11	70.95 ± 1.69***
***S. trifolia* (♀)**						
CP only	15	455.40 ± 39.62	16.48 ± 1.25	3.09 ± 0.37	80.44 ± 1.15	96.33 ± 0.43
HP only	15	432.67 ± 55.63	38.46 ± 2.39	61.15 ± 2.40	0.39 ± 0.22	0.64 ± 0.37
Mix (adequate)	15	423.20 ± 83.70	23.75 ± 1.64	12.64 ± 1.99	63.61 ± 2.61	83.29 ± 2.56***
Mix (inadequate)	15	383.47 ± 82.64	69.98 ± 3.32	6.36 ± 0.71	23.66 ± 2.87	78.05 ± 1.60***
Half-and-half	15	436.67 ± 78.51	19.00 ± 1.88	20.25 ± 1.05	60.75 ± 1.86	74.94 ± 1.21***

Values are means ± SE. N, the number of aggregate fruits sampled. One-sample t-test comparing the proportion of filled seeds sired by S. pygmaea or S. trifolia with the expected proportion (50%), ***p < 0.001. Total seeds (total number of filled seeds and unfilled seeds). CP, conspecific pollen; HP, heterospecific pollen. Hand-pollination treatment: CP only (pure conspecific pollen), HP only (pure heterospecific pollen), Mix (1:1 mixture of CP and HP. Adequate means that the total number of pollen grains were approximately 5–10 times the number of carpels; inadequate means the total number of pollen grains were approximately equal to the number of carpels), half-and-half (one half of the gynoecium was pollinated with adequate CP and the other half with HP).

## Discussion

### Heterospecific pollen can fertilize ovules, resulting in interspecific seed discounting

Pollen deposition from closely related species can cause ovule wastage in congeners, which is a fitness cost termed interspecific seed discounting (Burgess et al., 2008) and has been observed in numerous plant species (Fishman and Wyatt, 1999; Arceo-Gomez and Ashman, 2011; Nishida et al., 2014; Baek et al., 2016; Takemori et al., 2019; Hashimoto et al., 2021). Similar results have been found in the present study. Here, the seeds produced by interspecific crosses between *S. pygmaea* and *S. trifolia* contained partially developed embryos and could not germinate, demonstrating the existence of a post-fertilization isolation mechanism between the two species. This also explains why hybrid plants have not been observed in nature despite the widespread coexistence of these species. A previous study on *S. pygmaea* and *S. trifolia* demonstrated that interspecific pollen tubes could pass through the basal opening to enter the ovules of other carpels. Still, the authors did not evaluate whether HP can successfully complete fertilization (Lyu et al., 2017). In this study, we developed molecular techniques to investigate whether sterile embryos produced by interspecific pollination between two *Sagittaria* species result from heterospecific gamete fusion, confirming that interspecific pollen could enter the ovule and successfully fertilize the egg cells.

According to previous studies on plant fertilization, there exists a fertilization recovery phenomenon and polytubey block in flowering plants (Beale et al., 2012; Kasahara et al., 2012; Yu et al., 2021). When the first pollen tube fails to fertilize, the synergid cell can actively rescue fertilization by attracting a second functional pollen tube, using this fertilization recovery mechanism to maximize the successful seed set. However, once the first pollen tube successfully achieved double fertilization, the other pollen tubes are prevented from entering the ovule micropyle. Hence, we speculate that when HP tubes enter the ovules and fertilize successfully, CP tubes will not be able to fertilize the same ovule; at this time, the extragynoecial compitum, which allows HP tubes to grow from one to another carpel, may increase the number of wasted ovules, magnifying the risk of interspecific reproductive interference.

Although the hybrid seeds in our study showed abnormal embryo development, the morphology and size of hybrid fruits were similar to those of fruits produced from conspecific pollination. This result is similar to those observations for *Mimulus guttatus* and *M. nudatus*, as both self-fertilized and hybrid fruits typically exhibited an increase in size. However, the self-fertilized seeds were round, whereas those produced by interspecific crosses were shriveled and hardly germinated (Oneal et al., 2016). Hybrid fruits that are typical in shape but not in seed characteristics have also been found in other species with interspecific pollination, such as *Oryza*, *Solanum* section *Lycopersicon*, *Nemesia*, *Nicotiana*, and *Phacelia* (Datson et al., 2006; Glass and Levy, 2011; Ishikawa et al., 2011; Baek et al., 2016; He et al., 2020). It implies that normally expanding fruits in nature may also contain some inviable seeds that arise from interspecific pollination. Thus, reproductive interference caused by HP deposition in nature may be more severe than that currently recognized.

### Conspecific pollen advantage can mitigate the potential negative effects of interspecific pollen deposition

The extragynoecial compitum has the potential to magnify the risks of interspecific pollen deposition; however, our results suggest that the potential negative effects caused by HP are mitigated due to the CP advantage. In the CP and HP (1:1) mixed pollination experiment, we obtained more conspecific seeds (filled seeds) than expected (50%) based on the pollen ratio applied, indicating that CP has a seed-siring advantage over HP. In the half-and-half pollination treatment, we harvested 70.95% (*S. pygmaea*) and 78.05% (*S. trifolia*) of the filled seeds which were significantly higher than the proportion of carpels pollinated with CP to total carpels. It indicated that the CP tube could occupy ovules via the extragynoecial compitum that should be occupied by the HP. As CP has an advantage over HP, the extragynoecial compitum provides more opportunities for CP to occupy ovules, thereby alleviating the potential reproductive interference caused by HP and ensuring the reproductive success of the plants. It supports the view that intercarpellary pollen-tube growth mediated by the extragynoecial compitum in apocarpous angiosperms provides reproductive assurance under conditions of unpredictable pollination (Huang, 2003).

We speculated that the CP advantage exhibited by *S. pygmaea* and *S. trifolia* is likely to result from the extended growth route, lower fertilization rates or retarded growth of HP tubes in the pistils. Previous studies on *S. trifolia* have found that the maximum elongation distance of an extragynoecial pollen tube can span three carpels in a gynoecium (Huang and Wang, 2017). Thus, extragynoecial pollen-tube growth causes the pollen tube to travel a longer distance to reach the ovule. As the HP tube tends to pass through the opening at the base of the carpel into other carpels for fertilization (Lyu et al., 2017), it travels a longer path to enter ovules than the CP tube. Therefore, the time it takes for the pollen tube to enter the ovule may correspondingly increase. Our delayed CP pollination treatments showed that when HP pollination was followed by CP pollination after 0.5–1 h, approximately 50% or > 50% of filled seeds were obtained. This result is consistent with the finding that the HP tube was delayed in reaching the ovules by approximately 1 h due to longer routes (Lyu et al., 2017). Furthermore, based on Table 2, We could find that the HP pollination fertilized ovules and formed unfilled seeds; on the other hand, HP pollination led to a high proportion of “carpels without seeds” (the forth column in Table 2) which may suggest that the fertilization rate is lower after HP pollination as compared to CP pollination. Under this condition, the ovules could be fertilized by CP via the intercarpellary growth of pollen tubes. The results from the half-and-half pollination treatment support this hypothesis.

It is unclear whether there is a difference in the pollen tube growth rate between the species as HP tubes take longer to enter the ovule than CP tubes, and they also travel a longer route. A statistical analysis of pollen tube growth in 6,599 carpels at different stages after interspecific pollination between *S. pygmaea* and *S. trifolia* showed that the CP and HP tubes reached the vicinity of the ovary at approximately 1 h after pollination (Lyu et al., 2017). Therefore, the growth rate of pollen tubes in the early stages may not differ. However, pollen-tube growth rates may change with time in some species, such as in *Iris brevicaulis* and *I. fulva* (Emms et al., 1996). Differences in pollen tube growth patterns between CP and HP tubes may be due to the presence of species-specific substances in the pistil tissue that attract pollen tubes for directional growth and accelerate CP tubes enter ovules. A previous study on *Arabidopsis thaliana* showed that the species-specific female AtLURE1 peptides and their male counterparts—the PRK6 receptors—facilitate and maintain reproductive isolation by accelerating CP tubes to penetrate the septum and grow toward the ovules (Zhong et al., 2019). Similar species-specific substances might also be present in the pistil tissues of *Sagittaria* species, and this molecular mechanism deserves further investigation.

In apocarpous species, the extragynoecial compitum formed by the incomplete fusion of the carpel margins is the basis for the intercarpellary growth of pollen tubes. Its benefits in terms of reproductive assurance have received research attention. However, in the present study, we used *S. pygmaea* and *S. trifolia* as experimental materials to confirm that the incomplete fusion of the carpel margins allows HP tubes to enter the ovules and fertilize the egg cells, causing interspecific seed discounting. Our results also emphasize the importance of CP advantage, which is mediated by the extragynoecial compitum and mitigates the potential adverse effects of interspecific reproductive interference. Closely related species often share pollinators, especially in sympatric distributions (Mitchell et al., 2009; Runquist, 2012; Huang and Shi, 2013; Mizusawa et al., 2014; Tokuda et al., 2015; Katsuhara et al., 2019). Therefore, it is worthwhile investigating whether interspecific pollen transfer causes widespread reproductive interference and whether the CP advantage can mitigate the negative effect of HP deposition. *Sagittaria* plants in sympatric distributions, as well as exotic species such as *S. graminea*, have been found to hybridize with native plants (*S. trifolia*) (Zhang, 2014). Further studies on this genus could help better understand the prevalence of CP advantage mediated by the extragynoecial compitum and its role in maintaining species formation and stability.

## Conclusion

In this study, we found that although fertile hybrid progenies could not be produced between *S. pygmaea* and *S. trifolia*, the pollen tubes of both species could germinate and grow into ovules during interspecific pollination, producing the aggregated fruits consisting of normal-looking achenes. In addition, the molecular identification of partially developed embryos revealed that interspecific pollen could successfully fertilize ovules, indicating that the extragynoecial compitum has the potential to magnify the negative effects of HP. However, our hand-pollination experiments showed that CP has a seed-siring advantage over HP and that CP tubes can occupy more ovules through the extragynoecial compitum, thus mitigating the potential adverse effects of interspecific pollen deposition.

## Data availability statement

The original contributions presented in this study are included in the article/Supplementary Material, further inquiries can be directed to the corresponding author/s.

## Author contributions

X-FW and C-HF conceived and designed the experiments. C-HF, S-ST, S-HS, JD, and X-YW performed the experiments. C-HF analyzed the data and wrote a preliminary version of the manuscript. SW, W-QL, and X-FW revised the manuscript. All authors read and approved the manuscript.

## Conflict of interest

The authors declare that the research was conducted in the absence of any commercial or financial relationships that could be construed as a potential conflict of interest.

## Publisher’s note

All claims expressed in this article are solely those of the authors and do not necessarily represent those of their affiliated organizations, or those of the publisher, the editors and the reviewers. Any product that may be evaluated in this article, or claim that may be made by its manufacturer, is not guaranteed or endorsed by the publisher.
