# Supplementary material for: Conspecific pollen advantage mediated by the extragynoecial compitum and its potential to resist interspecific reproductive interference between two Sagittaria species
Source: Front Plant Sci. 2022 Jul 22;13:956193. doi: 10.3389/fpls.2022.956193 (PMC9354020; doi:10.3389/fpls.2022.956193)
Supplement: Supplementary file 1 [file Image_1.PDF]

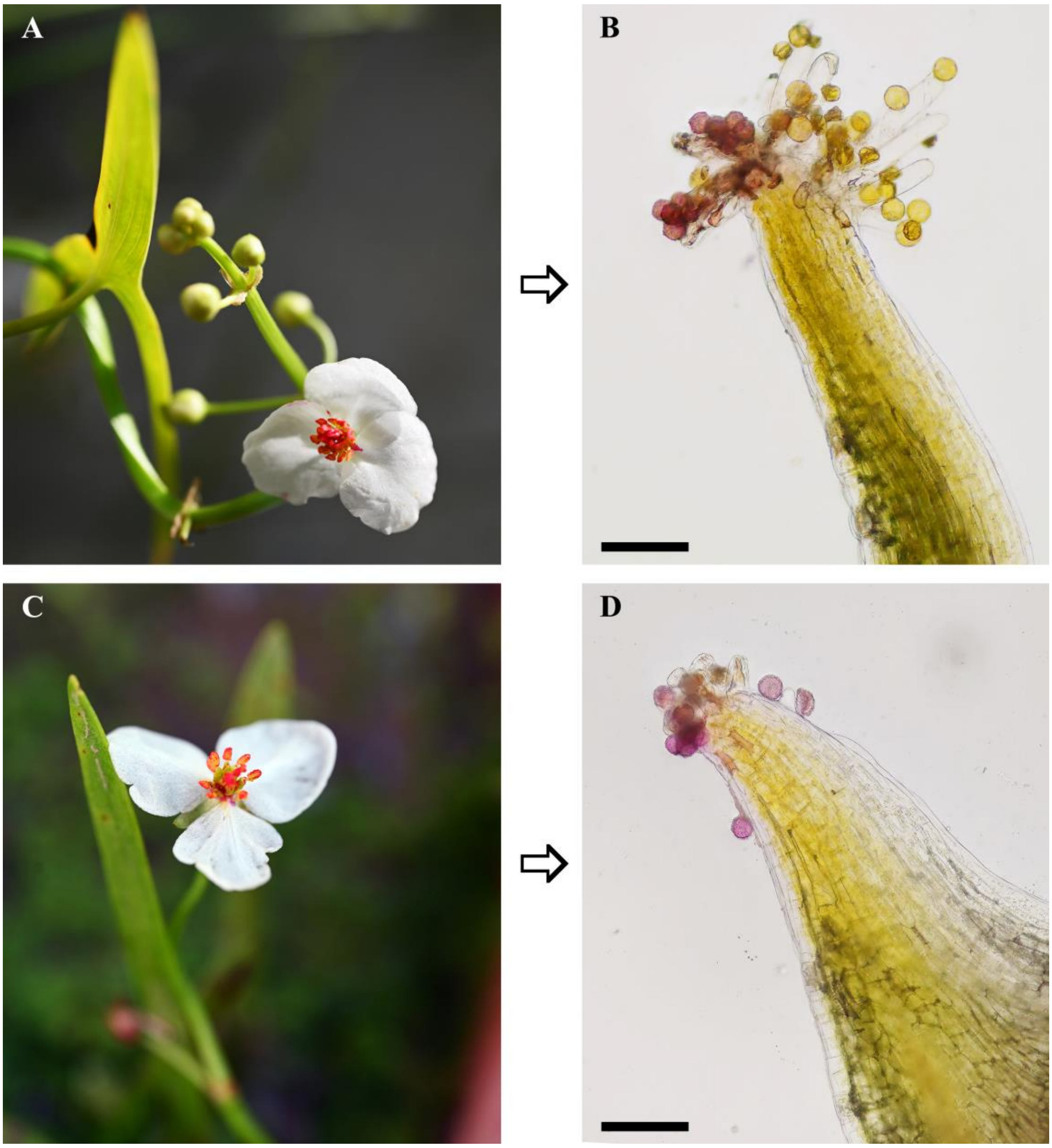

Supplementary Figure S1. Stained heterospecific pollen was detected on the stigma of *Sagittaria pygmaea* and *S. trifolia* female flowers in the mixed-species arrays. **(A)** Safranin-stained male flowers of *S. trifolia*. **(B)** *S. trifolia* safranin-stained red pollen grains were transferred to the stigma of *S. pygmaea*. **(C)** Safranin-stained male flowers of *S. pygmaea*. **(D)** Safrain-stained red pollen grains of *S. pygmaea* were transferred to the stigma of *S. trifolia*. Scale bar:100 µm.
