# Supplementary material for: Conspecific pollen advantage mediated by the extragynoecial compitum and its potential to resist interspecific reproductive interference between two Sagittaria species
Source: Front Plant Sci. 2022 Jul 22;13:956193. doi: 10.3389/fpls.2022.956193 (PMC9354020; doi:10.3389/fpls.2022.956193)
Supplement: Supplementary file 2 [file Image_2.PDF]

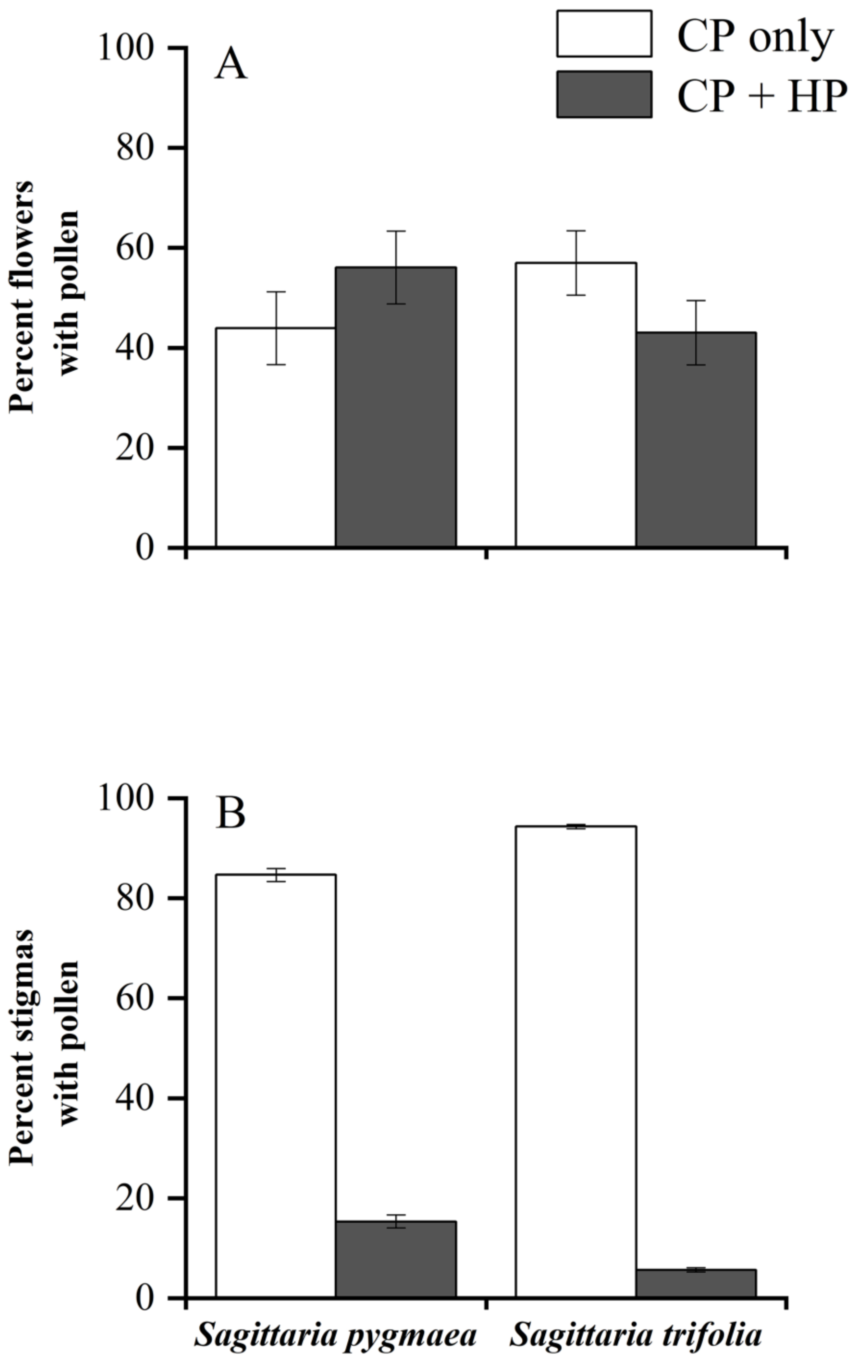

Supplementary Figure S2 Conspecific and heterospecific pollen deposition on *Sagittaria pygmaea* and *S. trifolia* in the mixed-species arrays. **(A)** The percentage of *S. pygmaea* and *S. trifolia* female flowers with or without the presence of interspecific pollens. **(B)** The percentage of *S. pygmaea* and *S. trifolia* stigmas with interspecific pollen or without interspecific pollen present. Data are represented as mean ± SE. CP; conspecific pollen, HP; heterospecific pollen.
