# Supplementary material for: Conspecific pollen advantage mediated by the extragynoecial compitum and its potential to resist interspecific reproductive interference between two Sagittaria species
Source: Front Plant Sci. 2022 Jul 22;13:956193. doi: 10.3389/fpls.2022.956193 (PMC9354020; doi:10.3389/fpls.2022.956193)
Supplement: Supplementary file 3 [file Table_1.docx]

**Supplementary Table S1.** Summary of aggregate fruits, achenes, and seeds characteristics for conspecific and interspecific crosses.

|  | *S. pygmaea* × *S. pyamaea* (N) | *S. pygmaea* × *S. trifolia* (N) | *t* | *P* | *S. trifolia × S. trifolia* (N) | *S. trifolia × S. pygmaea* (N) | *t* | *P* |
| --- | --- | --- | --- | --- | --- | --- | --- | --- |
| Average aggregate fruit  length (mm) | 9.16 ± 0.22 (30) | 8.87 ± 0.19 (30) | -0.988 | 0.327 | 13.23 ± 0.24 (36) | 13.35 ± 0.18 (37) | 0.424 | 0.673 |
| Average aggregate fruit  width (mm) | 8.77 ± 0.23 (30) | 8.45 ± 0.19 (30) | -1.071 | 0.289 | 12.52 ± 0.21 (36) | 12.46 ± 0.13 (37) | -0.234 | 0.816 |
| Average aggregate fruit  height (mm) | 7.41 ± 0.16 (30) | 7.20 ± 0.15 (30) | -0.972 | 0.335 | 10.22 ± 0.13 (36) | 10.05 ± 0.13 (37) | -0.961 | 0.340 |
| Average achene length (mm) | 4.83 ± 0.09 (30) | 4.29 ± 0.07 (30) | -4.664 | < 0.001 | 3.93 ± 0.06 (60) | 3.97 ± 0.03 (60) | 0.634 | 0.527 |
| Average achene width (mm) | 2.89 ± 0.07 (30) | 2.84 ± 0.05 (30) | -0.562 | 0.576 | 3.08 ± 0.05 (60) | 3.10 ± 0.03 (60) | 0.293 | 0.770 |
| Average seed length (mm) | 1.81 ± 0.05 (30) | 1.69 ± 0.04 (30) | -1.864 | 0.067 | 1.78 ± 0.01 (30) | 1.52 ± 0.03 (30) | -7.341 | < 0.001 |
| Average seed width (mm) | 0.97 ± 0.03 (30) | 0.88 ± 0.03 (30) | -2.350 | 0.022 | 1.04 ± 0.01 (30) | 0.85 ± 0.02 (30) | -8.569 | < 0.001 |

All crosses are female × male. N, the number of aggregate fruits sampled. Values are means ± SE. Statistical analyses by independent samples *t*-test.
