# Supplementary material for: Conspecific pollen advantage mediated by the extragynoecial compitum and its potential to resist interspecific reproductive interference between two Sagittaria species
Source: Front Plant Sci. 2022 Jul 22;13:956193. doi: 10.3389/fpls.2022.956193 (PMC9354020; doi:10.3389/fpls.2022.956193)
Supplement: Supplementary file 4 [file Table_2.docx]

**Supplementary Table S2.** Variable sites of ITS sequences obtained from *Sagittaria pygmaea*, *S. trifolia*, and their offspring from different pollination treatments.

| **The sources of DNA** | **Locus number and base type** | | | | | | | | | | | | | | | | | | | | | | | | | | | | | | | |
| --- | --- | --- | --- | --- | --- | --- | --- | --- | --- | --- | --- | --- | --- | --- | --- | --- | --- | --- | --- | --- | --- | --- | --- | --- | --- | --- | --- | --- | --- | --- | --- | --- |
|  | **ITS1** | | | | | | | | | | **5.8S** | | | **ITS2** | | | | | | | | | | | | | | | | | | |
|  | 1  3 | 5  1 | 6  7 | 1  2  0 | 1  7  2 | 1  8  8 | 2  0  1 | 2  0  2 | 2  2  9 | 2  4  9 |  | 3  2  4 |  | | 4  3  4 | 4  5  9 | 4  8  7 | 5  1  8 | 5  1  9 | 5  6  3 | 5  6  9 | 5  7  0 | 5  7  1 | 5  7  2 | 5  7  3 | 5  7  4 | 5  7  5 | 5  7  6 | 5  9  5 | 6  3  3 | 6  5  6 |  |
| **Leaf** | | | | | | | | | | | | | | | | | | | | | | | | | | | | | | | | |
| P1-P5 | T | T | G | A | T | C | G | C | C | A |  | A |  | | T | T | G | G | T | A | C | C | C | T | T | G | G | G | T | C | A |  |
| T1-T5 | C | C | C | T | C | T | A | T | T | G |  | T |  | | C | C | T | T | G | G | G | G | A | C | - | A | T | T | C | A | G |  |
| **Filled seeds** | | | | | | | | | | | | | | | | | | | | | | | | | | | | | | | | |
| PP1-PP10 | T | T | G | A | T | C | G | C | C | A |  | A |  | | T | T | G | G | T | A | C | C | C | T | T | G | G | G | T | C | A |  |
| PPT1-PPT16 | T | T | G | A | T | C | G | C | C | A |  | A |  | | T | T | G | G | T | A | C | C | C | T | T | G | G | G | T | C | A |  |
| TT1-TT10 | C | C | C | T | C | T | A | T | T | G |  | T |  | | C | C | T | T | G | G | G | G | A | C | - | A | T | T | C | A | G |  |
| TTP1-TTP10 | C | C | C | T | C | T | A | T | T | G |  | T |  | | C | C | T | T | G | G | G | G | A | C | - | A | T | T | C | A | G |  |
| **Unfilled seeds** | | | | | | | | | | | | | | | | | | | | | | | | | | | | | | | | |
| PT1-PT22 | **Y** | **Y** | **S** | **W** | **Y** | **Y** | **R** | **Y** | **Y** | **R** |  | **W** |  | | **Y** | **Y** | **K** | **K** | **K** | **R** | **S** | **S** | **M** | **Y** | **-/T** | **R** | **K** | **K** | **Y** | **M** | **R** |  |
| PT23 | **Y** | **Y** | **S** | **W** | **Y** | **Y** | **R** | **Y** | **Y** | **R** |  | **W** |  | | **Y** | **Y** | G | G | T | A | C | C | C | T | T | G | G | G | T | C | A |  |
| PT24 | T | T | G | A | **Y** | **Y** | **R** | **Y** | **Y** | **R** |  | **W** |  | | **Y** | **Y** | **K** | **K** | **K** | **R** | **S** | **S** | **M** | **Y** | **-/T** | **R** | **K** | **K** | **Y** | **M** | **R** |  |
| PT25 | **Y** | **Y** | **S** | **W** | T | C | G | C | C | A |  | A |  | | T | T | G | G | T | A | C | C | C | T | T | G | G | G | T | C | A |  |
| PT26 | T | T | G | A | T | C | G | C | C | A |  | **W** |  | | **Y** | **Y** | **K** | **K** | **K** | **R** | **S** | **S** | **M** | **Y** | **-/T** | **R** | **K** | **K** | **Y** | **M** | **R** |  |
| PT27 | T | T | G | A | T | C | G | C | C | A |  | A |  | | T | T | G | **K** | **K** | **R** | **S** | **S** | **M** | **Y** | **-/T** | **R** | **K** | **K** | **Y** | **M** | A |  |
| PT28-PT29 | T | T | G | A | T | C | G | C | C | A |  | A |  | | **Y** | **Y** | **K** | **K** | **K** | **R** | **S** | **S** | **M** | **Y** | **-/T** | **R** | **K** | **K** | **Y** | **M** | **R** |  |
| PT30 | T | T | G | A | T | C | G | C | C | A |  | A |  | | T | T | G | G | T | A | C | C | C | T | T | G | G | G | T | A | **R** |  |
| PT31 | T | T | G | A | T | C | G | C | C | A |  | A |  | | T | T | G | G | T | A | C | C | C | T | T | G | G | G | T | C | A |  |
| TP1-TP17 | **Y** | **Y** | **S** | **W** | **Y** | **Y** | **R** | **Y** | **Y** | **R** |  | **W** |  | | **Y** | **Y** | **K** | **K** | **K** | **R** | **S** | **S** | **M** | **Y** | **-/T** | **R** | **K** | **K** | **Y** | **M** | **R** |  |
| TP18 | C | C | C | T | C | T | A | T | T | G |  | T |  | | C | C | T | T | G | G | G | G | A | C | - | A | T | T | C | **M** | **R** |  |
| TP19 | **Y** | **Y** | **S** | **W** | **Y** | **Y** | **R** | **Y** | **Y** | **R** |  | **W** |  | | **Y** | **Y** | **K** | **K** | **K** | **R** | **S** | **S** | **M** | **Y** | **-/T** | **R** | **K** | **K** | **Y** | **M** | G |  |
| TP20 | **Y** | **Y** | **S** | T | C | T | A | T | T | G |  | T |  | | C | C | T | T | G | G | G | G | A | C | - | A | T | T | C | A | G |  |
| TP21-TP22 | C | C | C | T | C | T | A | T | T | G |  | T |  | | C | C | T | T | G | G | G | G | A | C | - | A | T | T | C | A | G |  |

Numbers refer to the nucleotide position in the complete alignment. -, gap. IUPAC ambiguity symbols are used to present polymorphisms (Y = C + T，S = C + G，K = T + G，W = A + T，M = A + C，R = A + G). P, *S. pygmaea*; T, *S. trifolia*.

Hand-pollination treaments: PP (*S. pygmaea*♀ × *S. pygmaea*♂), PPT (*S. pygmaea*♀ × *S. pygmaea*♂ × *S. trifolia*♂), TT (*S. trifolia*♀×*S. trifolia*♂), TTP (*S. trifolia*♀ × *S. trifolia*♂ × *S. pygmaea*♂), PT (*S. pygmaea*♀ × *S. trifolia*♂). TP (*S. trifolia*♀ × *S. pygmaea*♂).
